# Prediction of treatment failure in patients with glioblastoma with perfusion MRI and molecular biomarkers

**DOI:** 10.1093/noajnl/vdag119

**Published:** 2026-05-06

**Authors:** Riccardo Ludovichetti, Jordan Villiers, Gergely Bertalan, Nicolin Hainc, Andrea Bink, Emilie Le Rhun, Ulrike Held, Michael Weller, Zsolt Kulcsar, Ramona-Alexandra Todea

**Affiliations:** Department of Neuroradiology, Clinical Neuroscience Center, University Hospital and University of Zurich, Zurich, Switzerland; Epidemiology, Biostatistics and Prevention Institute, University of Zurich, Zurich, Switzerland; Department of Neuroradiology, Clinical Neuroscience Center, University Hospital and University of Zurich, Zurich, Switzerland; Department of Neuroradiology, Clinical Neuroscience Center, University Hospital and University of Zurich, Zurich, Switzerland; Department of Neuroradiology, Clinical Neuroscience Center, University Hospital and University of Zurich, Zurich, Switzerland; Department of Medical Oncology and Hematology, University Hospital Zurich, Zurich, Switzerland; Department of Neurorehabilitation, Clinic Lengg, Zurich, Switzerland (E.L.R.); Epidemiology, Biostatistics and Prevention Institute, University of Zurich, Zurich, Switzerland; Department of Neurology, Clinical Neuroscience Center, University Hospital and University of Zurich, Zurich, Switzerland; Department of Neuroradiology, Clinical Neuroscience Center, University Hospital and University of Zurich, Zurich, Switzerland; Department of Neuroradiology, Clinical Neuroscience Center, University Hospital and University of Zurich, Zurich, Switzerland; University of Basel, Basel, Switzerland; Division of Neuroradiology, Clinic of Radiology and Nuclear Medicine, University Hospital of Basel, Basel, Switzerland

**Keywords:** *MGMT*, glioblastoma, perfusion, treatment failure

## Abstract

**Background:**

Predicting early treatment failure in glioblastoma remains challenging. Given the median survival of 13.5 months, better prognostic biomarkers are needed. This study aimed to identify predictors of early treatment failure and their association with overall survival (OS).

**Methods:**

We performed a retrospective analysis of consecutive patients with newly diagnosed glioblastoma who underwent DSC- and DCE-MRI before surgery and radiochemotherapy. Treatment failure was defined as tumor progression per RANO 2.0 criteria within 6 months of surgery. Factors independently associated with outcome among clinical, MRI perfusion at the diagnosis and molecular parameters were identified using multivariable logistic regression models. OS was evaluated using a 180-day landmark analysis to avoid time-dependent bias.

**Results:**

Among 62 patients diagnosed between 01/2017 and 12/2021, 44 patients (71%) were non-responders and 18 patients (29%) responders. Elevated pretreatment rCBV and Ktrans were associated with treatment response (*P* < .05), with non-responders showing lower median rCBV (4.15 vs 5.90) and Ktrans (0.15 vs 0.17 min^−1^). A model, including age, perfusion metrics, and *MGMT* methylation status showed robust discrimination (AUC = 0.84, 95% CI: [0.73; 0.95]) to identify non-responders. Landmark analysis showed no evidence for early treatment failure itself to be associated with OS (*P* = .40), whereas *MGMT* promoter methylation status was associated with long-term survival and reduced hazard of death (HR: 0.31, 95% CI: 0.17-0.55; *P* < .001).

**Conclusion:**

Pretreatment MRI perfusion metrics may predict short-term treatment failure and may guide early experimental therapy, while the *MGMT* status remains the primary determinant of long-term survival.

Key Points

- Pretreatment MRI perfusion (rCBV, Ktrans) predicts treatment failure in glioblastoma.
- A model with age, perfusion, and MGMT methylation may discriminate non-responders (AUC = 0.84).
- *MGMT* methylation predicts long-term survival and reduced death hazard (HR: 0.31).

Importance of the Study

This study addresses the urgent need for early identification of treatment failure in glioblastoma, enabling optimized follow-up and timely therapeutic adjustments. Perfusion MRI biomarkers, particularly rCBV and Ktrans, may help identify non-responders early by distinguishing those with reduced vascularity and smaller extracellular-extravascular spaces, imaging features that correlate with higher tumor cellularity and predominantly unmethylated MGMT status. In contrast, patients who respond to treatment tend to have higher perfusion metrics and a methylated *MGMT* promoter status. MGMT methylation status emerged as the most significant factor associated with overall survival. By combining clinical (age), radiological (rCBV, Ktrans), and molecular (MGMT) parameters, a better discrimination was achieved between responders and non-responders compared to the clinico-radiological model alone. These findings support a multimodal prognostic framework integrating clinical, imaging, and molecular data, which could help guide more personalized treatment strategies in glioblastoma.

Glioblastoma is the most common and most aggressive primary malignant brain tumor in adults, with an incidence of about 3 per 100 000 persons per year^1^ and representing approximately 60% of malignant primary brain tumors.^2^^,^^3^ Despite decades of extensive research and clinical advances, prognosis remains poor: with current standard treatment, median survival is only 16-17 months in patients enrolled in clinical trials,^4^ 13.5 months in patients at the population level^5^ and fewer than 5% of patients live beyond 5 years.^6^^,^^7^ The standard of care consists of maximal safe resection of the contrast-enhancing tumor followed by radiotherapy with concomitant and maintenance temozolomide.^8^ Nevertheless, overall survival (OS) has improved only marginally.^9^ Predicting early treatment failure in GBM remains challenging, as conventional morphological MRI primarily reflects tumor size and the macroscopic extent of tumor infiltration. Given the persistently poor OS outcomes, improved biomarkers are urgently needed to enable patient stratification and identify early responders versus non-responders.

Molecular classification has transformed the understanding of GBM heterogeneity. Three primary transcriptomic subtypes of GBM have been described: receptor tyrosine kinase I (RTK I, proneural), receptor tyrosine kinase II (RTK II, classical), and mesenchymal (MES),^10^ but this classification has no impact in the clinic. Conversely, promoter methylation of O6-methylguanine-DNA methyltransferase (MGMT) predicts favorable response to temozolomide and longer survival.^11^^,^^12^

Tumor vascular biology can be effectively characterized through perfusion MRI, a powerful noninvasive technique. Dynamic susceptibility contrast MRI (DSC-MRI) estimates relative cerebral blood volume (rCBV), a surrogate of microvascular density,^13^ while dynamic contrast-enhanced MRI (DCE-MRI) provides parameters such as extravascular extracellular space volume (Ve), plasma volume (Vp), and the transfer constant (Ktrans), which reflect tumor perfusion, neoangiogenic activity, and capillary permeability.^14^ Together, these perfusion-derived MRI metrics provide quantitative insight into tumor hemodynamics and vascular function.

Glioblastoma heterogeneity is further reflected in the timing and dynamics of tumor progression, with a substantial subset of patients exhibiting early radiologic progression despite standard therapy. In this study, treatment failure is defined in patients exhibiting radiologic progression according to the RANO 2.0 criteria^15^ within 6 months after initial surgery. Early identification of these high-risk patients is critical, as it may support personalized surveillance strategies and inform timely consideration of alternative or experimental therapeutic approaches.

This study aimed to identify clinical, pretreatment radiological, and molecular predictors of treatment failure in glioblastoma, evaluate MRI-derived biomarkers and key molecular markers for predicting treatment failure and assess the relationship between treatment failure and OS.

## Methods

### Study Design and Participants

This is a retrospective, single-center study conducted at a tertiary-care academic neuro-oncology center including patients with glioblastoma diagnosed between January 2017 and December 2021. Inclusion criteria were (1) female and male adult patients who had (2) a contrast-enhanced (CE)-MRI at the diagnosis including DSC- and/or DCE-MRI studies and underwent (3) surgery followed by radiochemotherapy, (4) had a postoperative CE-MRI within 24 h after surgery and (5) serial follow-up MRIs to assess tumor progression and (6) available clinical and tumor molecular data. The exclusion criteria included incomplete or uninterpretable MRI follow-up. The study was approved by the ethics committee (BASEC number 2022-00913). Written informed consent was obtained from all participants or formally waived according to institutional regulations.

### Data Collection

Data were retrospectively extracted from the electronic medical records (PACS) of Basel University Hospital and included clinical variables (age at diagnosis, sex, and extent of resection), MRI perfusion biomarkers (rCBV, Ktrans, Ve, and Vp), and molecular features (IDH mutation, *MGMT* promoter methylation, EGFR amplification, and molecular subtypes: RTK I/II and mesenchymal).

Key temporal milestones, including dates of diagnosis, surgery, treatment initiation, radiological progression (multiple MRI follow-ups were available: post-surgery, pre-radiotherapy, monthly MRI for the first 3 months after combined radiochemotherapy, then each 3-6 months or earlier if clinical progression was suspected) death or last follow-up, were recorded to facilitate time-to-event survival analyses.

This study partially builds on a cohort previously described, including 62 patients included in both studies.^16^^,^^17^

### Imaging Acquisition

All MRI examinations were performed on a 3T scanner (MAGNETOM Skyra, Siemens Healthineers) using a standardized brain tumor protocol, including DWI, FLAIR, SWI, T2TSE, and pre- and post-contrast T1WI, as well as DSC- and DCE-MRI. A split-bolus technique was used, with the first contrast bolus administered for GRASP DCE-MRI serving as preload for subsequent DSC-MRI to minimize T1W-leakage effects. Methodology details for DCE- and DSC- MRI sequences were adapted from Vankan et al^16^ and Var et al.^17^ Treatment response on follow-up imaging was assessed on post-contrast T1WI according to RANO 2.0 criteria.^15^

### Imaging Assessment

All RANO assessments were performed by a board-certified neuroradiologist with 12 years of experience, blinded to clinical outcomes (RT). Perfusion post-processing was conducted using Olea Sphere (v3.0, Olea Medical), independently by 2 readers (RT—a board-certified neuroradiologist with 12 years of experience and a neuroradiology “resident in training,” VV) both blinded to treatment response at the time of post-processing. DCE-MRI parameters (Ktrans, Ve, Vp) were derived using an extended Tofts two-compartment model with automated arterial input function, while DSC-MRI analysis included leakage correction. Perfusion values were assessed in 2 manually defined ROIs (20-30 mm^2^) at the “hotspot” location on the color-coded maps. For rCBV measurements, regions affected by magnetic susceptibility artifacts were excluded. Normalization was performed by placing a third ROI in the normal-appearing white matter of the contralateral centrum semiovale.^16^^,^^17^ All imaging variables were extracted from pretreatment MRI acquired at diagnosis, prior to surgery or prior to further therapy.

### Outcome Measures

The primary variable of interest was treatment response (binary). Patients were classified as “non-responders” if progression occurred ≤6 months (≤180 days) after surgery. Patients remaining progression-free during these first 6 months were categorized as “responders.”

Secondary endpoints focused on long-term survival outcomes: survival was calculated from the date of surgery to the date of death from any cause. Surviving patients were censored at May 5, 2025 as administrative data cutoff date. This was the specific time-point where the final cross-sectional check of the medical records was performed to verify that all remaining patients included in the study were still alive. Therefore, all surviving patients were simultaneously right censored at this universal verification date.

### Statistical Analysis

Continuous variables were checked for distributional properties; right-skewed variables were log-transformed prior to analysis. Baseline clinical, imaging, and molecular characteristics were summarized using mean (SD), median [IQR], or frequencies (%), as appropriate. Baseline variables were descriptively summarized by response status. Group differences were evaluated using two-sample *t*-tests, Mann-Whitney *U* tests, or χ^2^ tests, as appropriate. Pearson correlation matrices were computed to evaluate associations and screen for multicollinearity among imaging biomarkers.

Early treatment failure was modeled using multivariable logistic regression. Predictors were grouped as clinical (age), imaging (rCBV, Ktrans), and molecular variables (*MGMT* promoter methylation). Two models were evaluated based on clinical reasoning and the study objective to evaluate novel biomarkers: Model 1 included age and perfusion metrics (rCBV and Ktrans), and Model 2 additionally incorporated *MGMT* methylation status. Model discrimination was assessed using the area under the receiver operating characteristic curve (AUC), and internal validation was performed using leave-one-out cross-validation to reduce overfitting (jackknife shrinkage).

OS was analyzed using Cox proportional hazards regression.

In this study, a landmark analysis at 180 days (6 months) was used to address 2 common methodological challenges in survival analysis, as well as an important clinical consideration:

Guarantee-time (immortal time) bias: the primary predictor is treatment response assessed at 6 months. By definition, a patient can only be classified as a “responder” if they survive progression-free for those first 6 months. If we measured OS from the baseline date of surgery, the responder group would inherently have a guaranteed survival time of at least 6 months, creating an artificial survival advantage.

Complete separation: Standard survival models face complete separation when no (or very few) deaths occur prior to the classification time-point, making it mathematically difficult for standard Cox models to estimate early survival curves properly.

Pseudoprogression: Clinically, choosing a 6-month landmark helped us avoid misclassifying patients who might experience pseudoprogression within the first 3 to 6 months following combined chemoradiation.

This approach only includes patients who survive past this landmark time-point and resets their “time zero” for survival estimation to this 6-month mark. This eliminates the immortal time bias, ensuring a rigorous, unbiased estimation of long-term survival outcomes between the 2 groups.

Given the exploratory nature of the study and the limited sample size, the number of predictors was restricted. Missing data (Supplementary Material and Supplementary Figure S1) were handled using missForest imputation, and analyses were conducted in accordance with the TRIPOD reporting guidelines.^18^

## Results

### Participants and Clinical Variables

**Figure 1.**
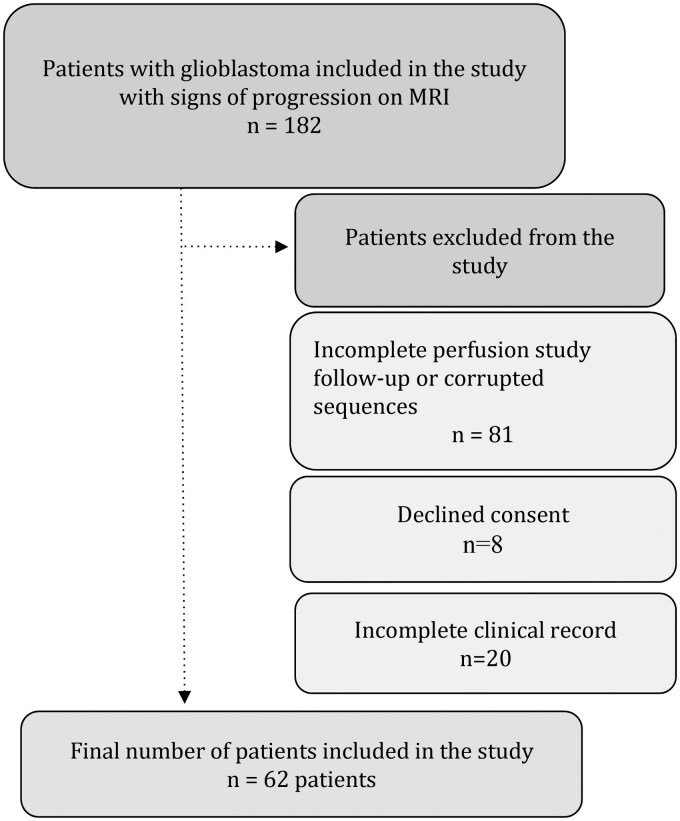
Flowchart of patients included in the study.

**Figure 2.**
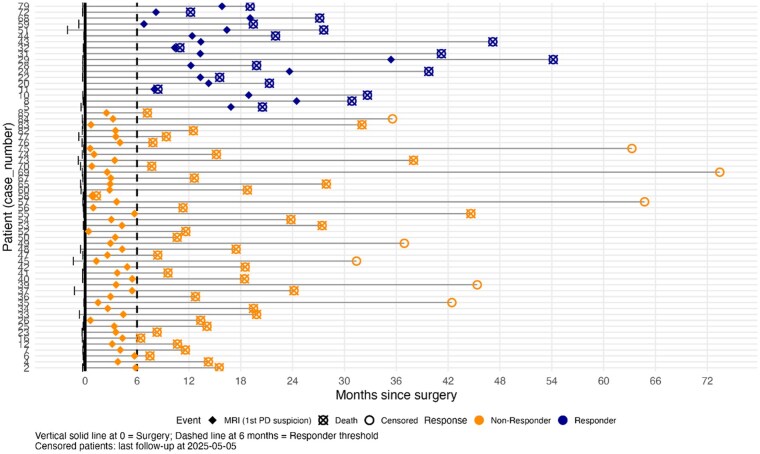
Patient timelines showing surgery (*t* = 0), timepoint of progression (MRI), and overall survival status. The dashed vertical line at 6 months represents the threshold for defining nonresponse. Yellow trajectories indicate patients with early progression (≤ 180 days), while blue trajectories indicate responders. Non-responders (Yellow): this group is characterized by early MRI progression events (diamonds) occurring before the 180-day mark. Visually, a cluster of mortality events (crossed squares) follows shortly after progression, indicative of an aggressive disease trajectory. Responders (Blue): by definition, these patients remained progression-free for the first 6 months. The plot highlights a subset of long-term survivors within this group, with follow-up durations extending beyond 48 months.

A total of 62 patients with newly diagnosed IDH-wild-type glioblastoma met the inclusion criteria and were included in this retrospective study (Figure 1). The cohort was predominantly male (*N* = 39, 62.9%), with a mean age at diagnosis of 60 years (SD = 11.6). Early treatment failure was common: 44 patients (71%) were classified as non-responders (radiographic progression ≤6 months) whereas 18 patients (29%) were responders (Figure 2).

Non-responders were older at diagnosis than responders (mean 61 vs 55 years), with the age distribution in non-responders shifted toward higher values and showing a higher median compared with responders (Supplementary Figure S2). Responders demonstrated a broader age range with a larger representation of younger patients. Females were numerically more frequently represented among responders (8/18, 44%) than non-responders (15/44, 34%), whereas males were more often non-responders (29/44, 66%) than responders (10/18, 56%).

All patients underwent surgery for diagnostic confirmation. Extent of resection was comparable between groups, with gross total resection achieved in 27.3% of non-responders and 33.3% of responders. Baseline characteristics are summarized in Table 1.

**Table 1.** Baseline characteristics of 62 glioblastoma patients

Level		Overall	Non-responder	Responder
*n*		62	44	18
Age at diagnosis (mean [SD])		59.48 (11.56)	61.43 (10.25)	54.72 (13.42)
Sex (%)	Female	23 (37.1)	15 (34.1)	8 (44.4)
	Male	39 (62.9)	29 (65.9)	10 (55.6)
Extent of resection (%)	Biopsy	6 (9.7)	3 (6.8)	3 (16.7)
	Complete resection (GTR)	18 (29.0)	12 (27.3)	6 (33.3)
	Subtotal resection (STR)	35 (56.5)	26 (59.1)	9 (50.0)
	Complete re-resection	2 (3.2)	2 (4.5)	0 (0.0)
	Partial re-resection	1 (1.6)	1 (2.3)	0 (0.0)
Post-operative radiotherapy alone (%)	No	11 (17.7)	7 (15.9)	4 (22.2)
	Yes	51 (82.3)	37 (84.1)	14 (77.8)
Post-operative treatment Radiochemotherapy and concomitant temozolomide (%)	No	5 (8.1)	5 (11.4)	0 (0.0)
	Yes	57 (91.9)	39 (88.6)	18 (100.0)
Post-operative radiotherapy and concomitant temozolomide followed by maintenance temozolomide (%)	No	11 (17.7)	11 (25.0)	0 (0.0)
	Yes	51 (82.3)	33 (75.0)	18 (100.0)

### MRI Variables

Across the cohort, the median rCBV was 4.23 (IQR: 2.63-6.15). Non-responders exhibited lower median values for the perfusion and permeability metrics than responders. Median rCBV was 4.15 (IQR: 2.83-5.30) in non-responders compared with 5.90 (IQR: 2.30-7.88) in responders (*P* = .49). Median Ktrans was also numerically lower in non-responders (0.15 min^−1^ [0.10-0.22] vs 0.17 min^−1^ [0.12-0.59]) (*P* = .18).

**Figure 3.**
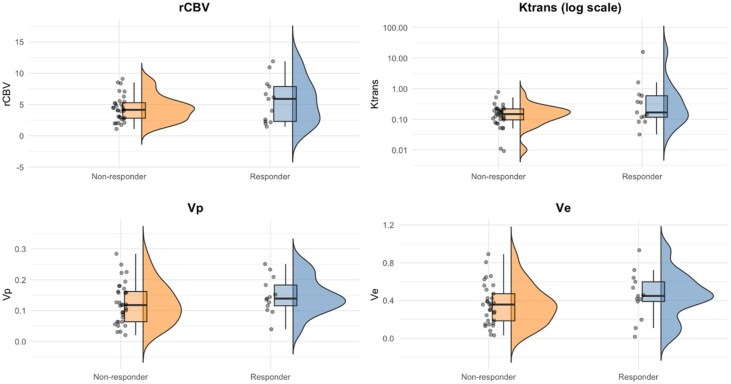
Raincloud plots showing the distribution of MRI biomarkers (rCBV, Ktrans, Vp, Ve) at the diagnosis stratified by response status. Responders tend to show elevated median values for rCBV compared to non-responders.

Similarly, Ve was lower in non-responders (0.36 mL/100 g [0.18-0.47]) compared with responders (0.45 mL/100 g [0.39-0.60]), and median Vp was modestly lower (0.12 mL/100 g [0.06-0.16] vs 0.14 mL/100 g [0.12-0.18]). Missing data for perfusion variables ranged from 25.8% to 27.4% (Table 2 and Figure 3).

**Table 2.** MRI biomarkers and molecular characteristics were descriptively summarized for responders and non-responders using median [IQR] for continuous variables and counts (%) for categorical variables

Overall		Non-responder	Responder	*P* value	
	62	44	18		Missing (%)
rCBV (median [IQR])	4.23 [2.63, 6.15]	4.15 [2.83, 5.30]	5.90 [2.30, 7.88]	.491	27.4
Ktrans (median [IQR])	0.15 [0.10, 0.23]	0.15 [0.10, 0.22]	0.17 [0.12, 0.59]	.180	25.8
Ve (median [IQR])	0.38 [0.19, 0.53]	0.36 [0.18, 0.47]	0.45 [0.39, 0.60]	.153	25.8
Vp (median [IQR])	0.12 [0.09, 0.17]	0.12 [0.06, 0.16]	0.14 [0.12, 0.18]	.176	25.8
*MGMT*_status = methylated (%)	29 (46.8)	18 (40.9)	11 (61.1)	.243	0.0
EGFR gene amplification = Yes (%)	20 (42.6)	13 (41.9)	7 (43.8)	1.000	24.2
GMC RTK I = Yes (%)	26 (41.9)	20 (45.5)	6 (33.3)	.552	0.0
GMC RTK II = Yes (%)	32 (51.6)	24 (54.5)	8 (44.4)	.658	0.0
GMC mesenchymal = Yes (%)	19 (30.6)	14 (31.8)	5 (27.8)	.992	0.0

Bivariate tests were performed to compare groups (Mann-Whitney *U* test for continuous variables, chi-square test for categorical variables).

Pearson correlation matrices were used to assess potential collinearity among the predictors and ensure biological consistency (Supplementary Figure S3). A strong positive correlation was observed between Ktrans and Ve (*r* = 0.83). Additionally, a moderate positive correlation was found between rCBV and Vp (*r* = 0.48). rCBV showed only weak negative correlations with both Ktrans (*r* = −0.19) and Ve (*r* = −0.25).

A reliability analysis was performed between 2 readers, and the intraclass correlation coefficient (ICC) was calculated for both DSC- and DCE-MRI parameters. The findings were as follows: ktrans = 0.79 (excellent reliability), Ve = 0.70 (good reliability), rCBV = 0.40 (fair reliability), and Vp = 0.02 (poor reliability).

### Molecular Variables

*MGMT* promoter methylation was more frequent among responders (11, 61.1%) than non-responders (18, 40.9%). EGFR amplification occurred at similar frequencies in responders and non-responders (43.8% vs 41.9%), with missing data in 14 cases (24.2%). RTK II was the predominant subtype in the overall cohort (51.6%), with no missing data (Table 2 and Figure 3).

### Model Development and Specification

Two multivariable logistic regression models were constructed to assess predictors of treatment failure. Model 1 included clinical and imaging variables (age, rCBV, and log [Ktrans]), while Model 2 additionally incorporated *MGMT* promoter methylation status. Internal validation was performed using coefficient shrinkage to mitigate overfitting. Model discrimination was assessed using the area under the receiver operating characteristic curve (AUC). Shrunken coefficients and performance metrics are reported in Supplementary Table S1.

**Figure 4.**
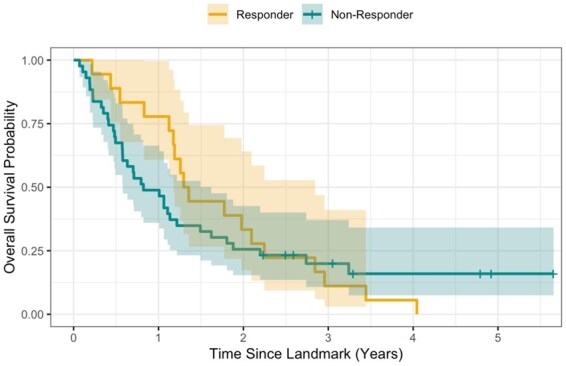
Kaplan-Meier estimates of overall survival conditional on surviving the first 6 months (landmark). Time 0 on the x-axis corresponds to 6 months post-surgery.

Time-to-event analyses were conducted using landmark Cox proportional hazards models at 180 days post-surgery. Landmark Model 1 (LM1) included clinical and imaging variables, and Landmark Model 2 (LM2) additionally incorporated MGMT status. Hazard ratios (log [HR]) with 95% confidence intervals were estimated, and model fit was compared using the Akaike information criterion (AIC). Results are presented in Supplementary Table S2 with OS curves estimated using the Kaplan-Meier method shown in Figure 4.

### Model Performance

Model 1 demonstrated good discrimination for predicting treatment failure (AUC = 0.80; 95% CI, 0.68-0.92). The inclusion of MGMT promoter methylation in Model 2 further improved discrimination (AUC = 0.85; 95% CI, 0.74-0.96) (Supplementary Table S1).

For survival analysis, LM2 showed improved model fit compared with LM1, reflected by a lower AIC (349.8 vs 363.7) (Supplementary Table S2). These findings indicate that the addition of molecular information provided incremental improvement in predicting both early treatment failure and OS.

## Discussion

This study aimed to identify clinical, radiological and molecular predictors of early treatment failure in IDH wild-type glioblastoma and their association with OS.

A model incorporating age, perfusion metrics (rCBV, Ktrans), and *MGMT* methylation status showed robust discrimination (AUC = 0.84, 95% CI: [0.73; 0.95]) for identifying non-responders. Landmark analysis showed no evidence for early treatment failure itself to be associated with OS (*P* = .40), whereas *MGMT* promoter methylation status was associated with long-term survival and reduced hazard of death (HR: 0.31, 95% CI: 0.17-0.55; *P* < .001).

### Predictor of Treatment Failure

Early treatment failure at 6 months was frequent (71%). This study results suggest that elevated pretreatment rCBV and Ktrans were associated with treatment response (*P* < .05). Non-responders tend to show lower median values for both rCBV ( 4.15 vs 5.90) and the transfer constant between the intravascular and the extracellular extravascular space (Ktrans 0.15 vs 0.17 min^−1^). While prior studies have consistently reported a correlation between higher perfusion metrics and increased tumor vascularity and aggressiveness^19-21^ our findings suggest a more complex relationship. Specifically, elevated perfusion might not only indicate a fast-growing and aggressive tumor but could also be associated with a more favorable response to treatment, possibly indicating a tumor subtype that is more amenable to therapy.^22^^,^^23^ These results raise the question of whether increased tumor perfusion at diagnosis could enhance drug delivery or potentially augment the effects of radiotherapy during the early phase of treatment.^24^ Additionally, responders exhibited higher values of extracellular-extravascular space (Ve), which may indicate less cellularity in tumors associated with better treatment outcomes. Given that Ktrans is influenced by both cerebral blood flow and the permeability of the blood-brain barrier, and that extracellular extravascular space is impacted by tumor cellularity (with more cellular tumors showing lower Ve), the strong positive correlation observed between Ktrans and Ve (*r* = 0.83) suggests that tumors with higher vascular permeability (as reflected by higher Ktrans) may also have greater extracellular space, likely due to lower cellularity. This relationship may imply that tumors with efficient blood flow and higher permeability might have a less densely packed cellular structure, facilitate greater contrast agent leakage, and potentially enhance drug delivery to the tumor microenvironment (Supplementary Figure S3).

Nevertheless, it is important to note that these conclusions should be viewed with some caution. While our data suggests a trend toward better outcomes in tumors with higher perfusion metrics, the conflicting results in the existing literature highlight the need for larger, more robust studies to validate these observations.

### Molecular Profiling and Model Performance

Clinical studies have consistently demonstrated that patients with methylated MGMT promoters benefit more from temozolomide-based chemoradiotherapy than those with unmethylated promoters, achieving longer progression-free and OS.^25^^,^^26^

This study further contributes to the literature by demonstrating that incorporating MGMT promoter methylation status enhances the discriminative power of predictive models. Multivariable logistic regression models combining clinical, imaging, and molecular variables showed robust discrimination between early non-responders and responders. The first model, which included age and perfusion metrics (rCBV and Ktrans), achieved an AUC of 0.80 (95% CI: 0.68-0.92), while adding MGMT promoter methylation in the second model increased performance to an AUC of 0.84 (95% CI: 0.73-0.95). Patients with methylated promoters were significantly less likely to experience early treatment failure.

Regarding molecular subtype distribution, no clear trend toward differential enrichment between non-responders and responders was observed. These findings suggest that RTK classification alone did not demonstrate a consistent descriptive trend toward early treatment response in this cohort. EGFR amplification was also similarly distributed across groups.

### Associations With Survival

Landmark analysis showed that MGMT methylation status was the dominant factor driving long-term survival (HR: 0.31, 95% CI: 0.17-0.55; *P* < .001), whereas early treatment failure was not independently associated with OS after accounting for molecular factors (*P* = .40).

Elevated rCBV and Ktrans reflected intrinsic tumor biology and vascular phenotype rather than being direct predictors of long-term survival.

Although the results of the study are compelling, there are a few limitations that should be considered when interpreting the results. First, the monocentric nature and relatively small cohort size limit the generalizability of our findings. Additionally, the use of manually defined regions of interest to extract quantitative perfusion parameters presents a methodological limitation. Although volumetric analysis could provide valuable insights, it was not feasible within the time constraints of routine clinical practice, where a large number of MRIs are reviewed daily. Furthermore, automated segmentation tools, which could potentially improve accuracy and reduce bias, are still in the process of being integrated into clinical settings and have not yet been widely validated. Our study focused on measuring perfusion parameters, such as rCBV, Ktrans, Ve, and Vp, as these parameters can be obtained within 10-20 min of postprocessing and are easily analyzed using our current tools. While missing data, particularly for imaging and molecular variables, could introduce potential bias, we mitigated this risk by applying the missForest imputation method, which effectively handles missingness without introducing significant bias. Finally, although our findings suggest interesting trends regarding the prognostic value of perfusion metrics such as rCBV and Ktrans, they should be interpreted cautiously given the existing literature. Larger, prospective studies are needed to validate these results and to better understand how vascular functionality and permeability influence glioblastoma response to therapy.

Despite these limitations, few studies have comprehensively integrated clinical variables, molecular alterations, and perfusion MRI metrics to predict treatment response in glioblastoma.^27-30^ The strengths of this study include the integration of multimodal data, use of quantitative imaging metrics, and internal validation despite a modest sample size. The findings support a hierarchical biomarker strategy, where perfusion MRI identifies patients at high risk of early treatment failure, guiding intensified monitoring or alternative experimental therapies, while molecular profiling remains critical for long-term prognostication. Early identification of non-responders through integrative models allows clinicians to proactively adjust therapy, including closer surveillance, intensified chemoradiotherapy, or clinical trial enrollment, ultimately improving outcomes and quality of life for high-risk patients.

In conclusion, the MRI perfusion biomarkers, particularly rCBV and Ktrans may provide valuable early predictors of treatment failure in glioblastoma patients, while the *MGMT* status remains the primary determinant of long-term survival. When combined they enhance personalized monitoring and risk stratification, helping to identify patients who may benefit from alternative or experimental therapies.

## Supplementary Material

vdag119_Supplementary_Data

## Data Availability

Data generated or analyzed during the study are available from the corresponding author by request.
