# Supplementary material for: Prediction of treatment failure in patients with glioblastoma with perfusion MRI and molecular biomarkers
Source: Neurooncol Adv. 2026 May 6;8(1):vdag119. doi: 10.1093/noajnl/vdag119 (PMC13200788; doi:10.1093/noajnl/vdag119)
Supplement: vdag119_Supplementary_Data [file vdag119_supplementary_data.docx]

**SUPPLEMENTARY MATERIAL**

**Material and methods**

**MRI acquisition protocol and postprocessing**

The dynamic sequences were acquired in one protocol with a split-bolus technique where the first contrast bolus injected for the GRASP DCE-MRI served as preload to correct for T1-weighted leakage effects in the consecutive DSC-MRI acquisition, with the purpose to avoid the underestimation of rCBV.

Initially, a 25 mL intravenous injection of NaCl (0.9% saline) is administered prior to the contrast agent. Baseline acquisition for the GRASP DCE-MRI sequence is performed for 20 seconds. Following this, 10 mL of Gadoteracid is injected at a rate of 2 mL/s, and dynamic DCE-MRI acquisition is conducted for a total duration of 04:23 minutes.

Subsequently, a 25 mL NaCl bolus is injected, and baseline acquisition for DSC-MRI is performed for 15 seconds. The second dose of Gadoteracid (10 mL) is then administered at a rate of 5 mL/s, with dynamic DSC-MRI acquisition continuing for 02:96 minutes. Finally, 30 mL of NaCl is reinjected to complete the protocol.

GRASP DCE-MRI uses a continuous acquisition of k-space with the following parameters: TR 4.09 ms, TE 1.92 ms, flip angle 12°, FOV 240 mm, matrix size 256 × 256, voxel size 1.5-mm isotropic, radial views 850, slice partial Fourier 6/8, bandwidth 400Hz/pixel, 4:23 min total acquisition. The dynamic images were reconstructed with a tRes of 4.3 seconds.

DSC-MRI was acquired using a T2*-weighted gradient-echo single-shot echoplanar imaging sequence with GRAPPA acceleration and motion correction. The sequence parameters included: TR 2000 ms, TE 35 ms, flip angle 60°, FOV 230 mm, matrix size 128 × 128 mm, slice thickness 6mm; voxel size 1.8 mm × 1.8 mm × 6.0 mm, bandwidth 1446 Hz/pixel; acquisition time 02:96 min.

The postprocessing of perfusion studies was done with Olea Sphere v3.0 (Olea Medical SAS, La Ciotat, France). The parameters were extracted from GRASP DCE-MRI using a two-compartment extended Tofts model with automated arterial input function at the level of the ICA.

For the DSC-MRI analysis, an arterial input function and a correction for contrast-agent leakage to extra-vascular space through the estimation of a leakage parameter (K2) were used.

**Statistical Analysis**

The appendices can be provided by request to the senior author.

**Missing Data**

Missing data were present across imaging and molecular variables (up to ~27% for perfusion MRI and ~24% for EGFR amplification). Missing values were imputed using the non-parametric missForest algorithm, which iteratively trains random forests on observed data to capture non-linear relationships without parametric assumptions. The pattern of missingness is summarized in Supplementary Figure S1.

**Prediction of treatment non-response (logistic regression)**

Model calibration was examined to verify the concordance between predicted probabilities and observed outcomes (Supplementary Figure S4. and S5.). The visual assessment using loess smoothing reveals that model 2 (bottom panel) achieves a calibration slope that aligns more closely with the ideal diagonal than model 1, particularly within the higher probability range. This improvement suggests that integrating molecular data enhances the reliability of risk predictions.

**FIGURES**


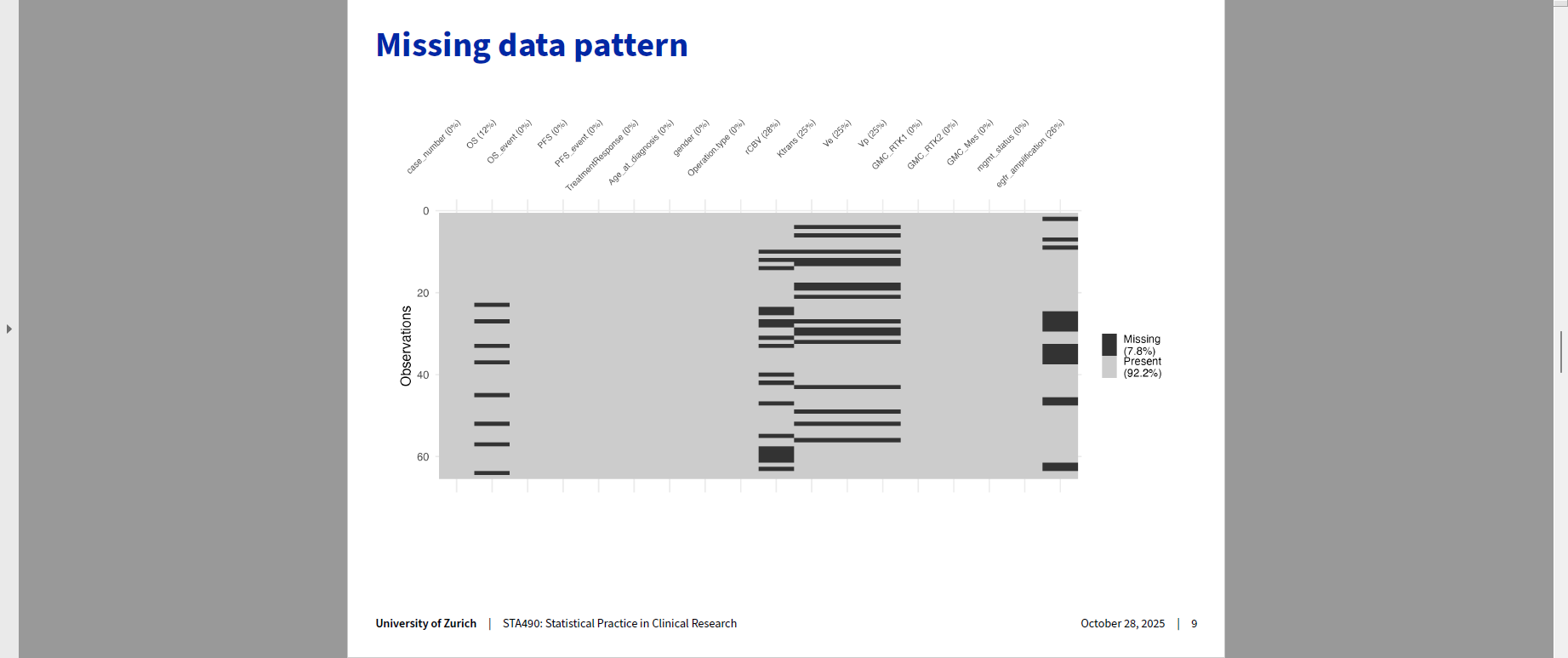


**S1.** Missing data pattern. Black bars indicate missing values; grey bars indicate present values. Clinical data (Age, Sex) are complete, while MRI biomarkers show approximately 26% missingness.


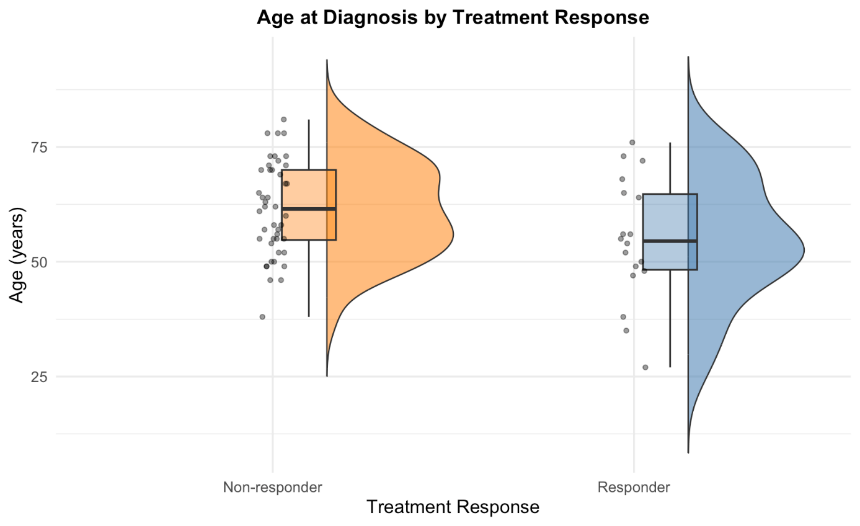


**S2. Age at diagnosis by treatment response.** Violin plots with embedded boxplots illustrate the distribution of age at diagnosis among non-responders and responders. Non-responders show a shift toward higher ages at diagnosis compared with responders, with a higher median and broader distribution. Individual data points are overlaid, demonstrating greater age variability among responders, while non-responders cluster more tightly around older ages.


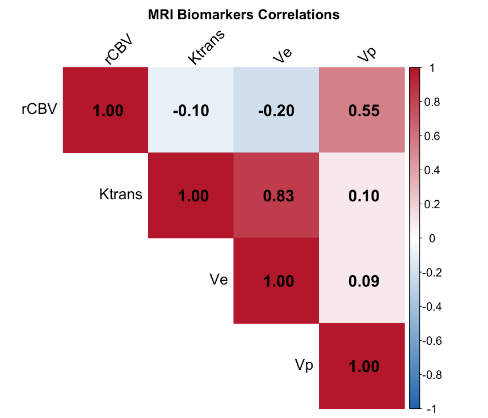


**S3.** Pearson correlation matrices. MRI-derived parameters showing a strong Ktrans- Ve association (r = 0.83).


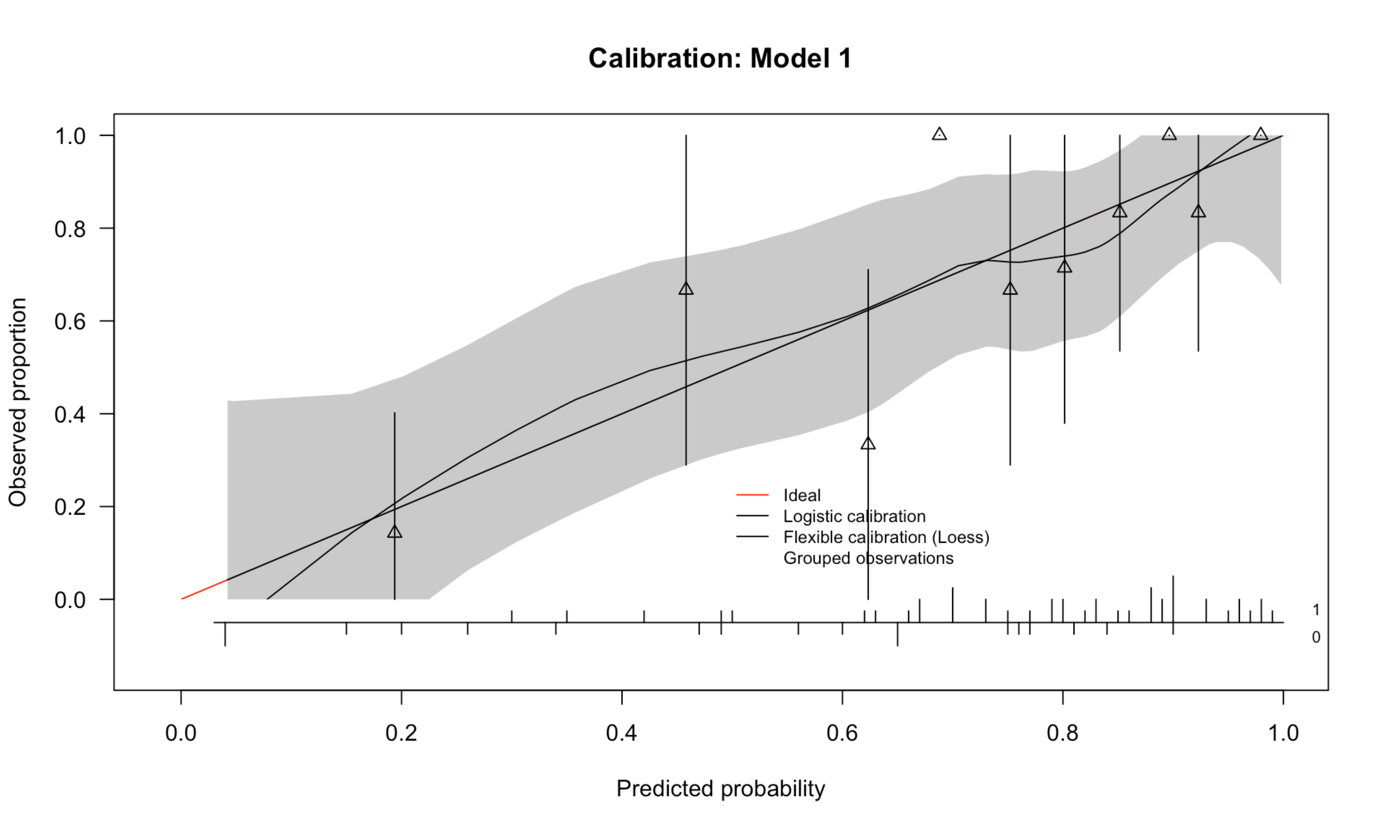


**S4.** Calibration Plot (Model 1)


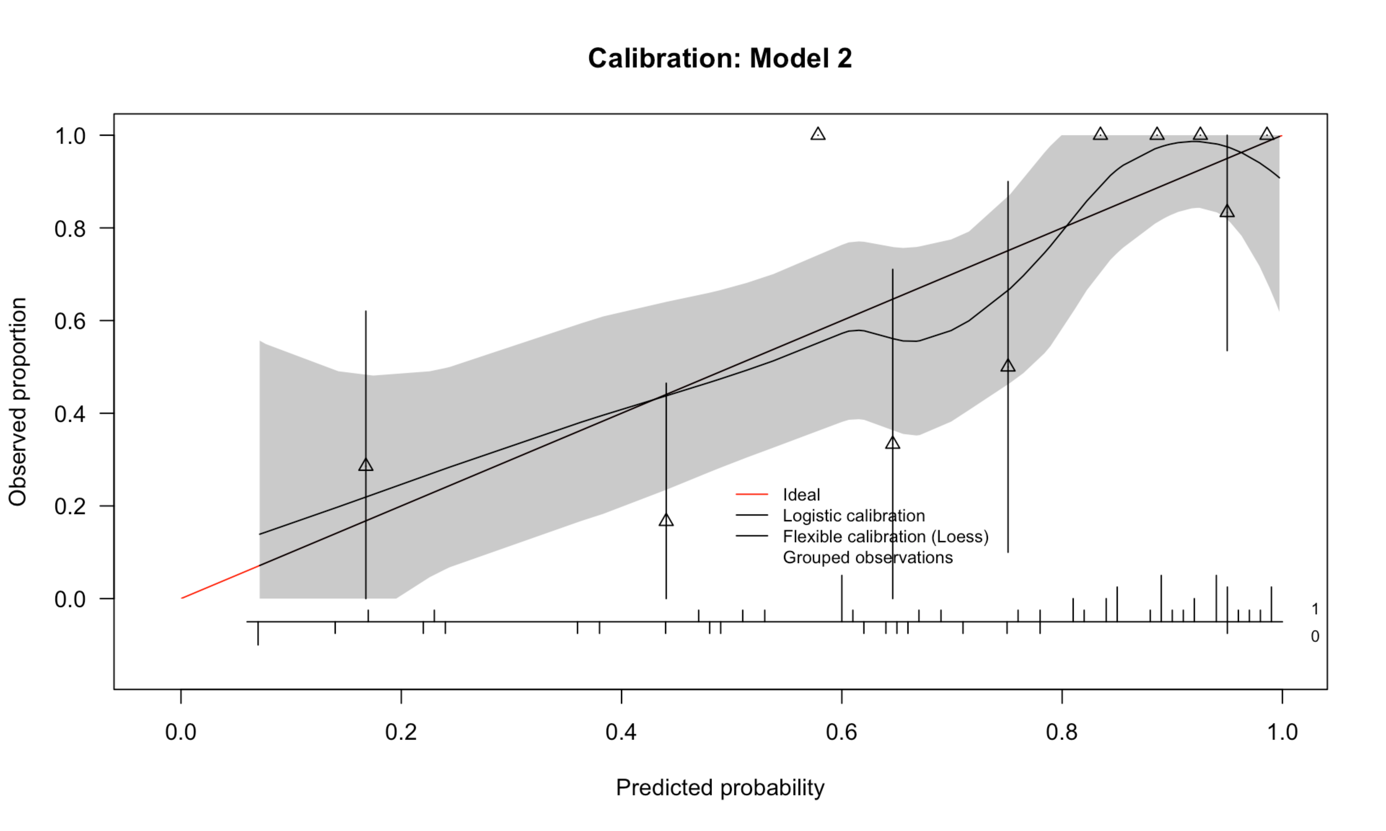


**S5.** Calibration Plot (Model 2)

**TABLES**

**Supplementary Table S1.** Multivariable Logistic Regression Models and Predictive Performance

| **Model** | **Predictor** | **Log Odds Ratio** | **95% CI** | ***P* Value** | **Shrinkage Factor** | **Shrunken Coefficient** | **AUC (95% CI)** |
| --- | --- | --- | --- | --- | --- | --- | --- |
| **Model 1** (Clinical + MRI) | Intercept | 1.22 | 0.51, 1.92 | <0.001 | — | 1.09 | 0.80  (0.68, 0.92) |
|  | Age | 0.04 | −0.02, 0.10 | 0.18 | 0.39 | 0.02 |  |
|  | rCBV | −0.40 | −0.73, −0.07 | 0.017 | 0.71 | −0.29 |  |
|  | log(Ktrans) | −1.13 | −1.99, −0.28 | 0.01 | 0.81 | −0.92 |  |
| **Model 2** (Clinical + MRI + Molecular) | Intercept | 1.92 | 0.75, 3.08 | 0.001 | — | 1.42 | 0.84  (0.73, 0.95) |
|  | Age | 0.03 | −0.03, 0.09 | 0.26 | 0.28 | 0.01 |  |
|  | rCBV | −0.45 | −0.79, −0.10 | 0.011 | 0.68 | −0.30 |  |
|  | log (Ktrans) | −1.19 | −2.02, −0.36 | 0.005 | 0.8 | −0.96 |  |
|  | *MGMT* (methylated) | −1.23 | −2.64, 0.18 | 0.088 | 0.51 | −0.62 |  |

AUC = area under the receiver operating characteristic curve; CI = confidence interval; log (Ktrans) = natural logarithm of the volume transfer constant derived from dynamic contrast-enhanced MRI, reflecting contrast transfer from blood plasma into the extravascular extracellular space; *MGMT* = O^6^-methylguanine–DNA methyltransferase (promoter methylation status); OR = odds ratio; rCBV = relative cerebral blood volume.

**Supplementary Table S2.** Landmark Cox Proportional Hazards Models and Model Comparison

| **Model** | **Predictor** | **log(HR)** | **95% CI** | ***P* Value** | **AIC** |
| --- | --- | --- | --- | --- | --- |
| **LM1** (Clinical + MRI) | Age | 0.01 | −0.02, 0.04 | 0.43 | 363.711 |
|  | rCBV | 0.06 | −0.06, 0.19 | 0.31 |  |
|  | log(Ktrans) | 0.2 | −0.09, 0.49 | 0.18 |  |
|  | Treatment failure | 0.27 | −0.36, 0.89 | 0.4 |  |
| **LM2** (Clinical + MRI + Molecular) | Age | 0.02 | −0.01, 0.04 | 0.26 | 349.744 |
|  | rCBV | 0.04 | −0.08, 0.17 | 0.49 |  |
|  | log (Ktrans) | 0.2 | −0.09, 0.49 | 0.18 |  |
|  | *MGMT* promoter methylation | −1.18 | −1.77, −0.59 | <0.0001 |  |
|  | Treatment failure | 0.23 | −0.45, 0.90 | 0.52 |  |

AIC = Akaike information criterion; CI = confidence interval; HR = hazard ratio; log(HR) = natural logarithm of the hazard ratio; log(Ktrans) = natural logarithm of the volume transfer constant derived from dynamic contrast-enhanced MRI, reflecting contrast transfer from blood plasma into the extravascular extracellular space; MGMT = O6-methylguanine–DNA methyltransferase (promoter methylation status); rCBV = relative cerebral blood volume.
